# Significance of a Tumor Mutation Burden Gene Signature with Prognosis and Immune Feature of Gastric Cancer Patients

**DOI:** 10.1155/2022/7684606

**Published:** 2022-06-08

**Authors:** Li Xiang, Chuan Lan

**Affiliations:** ^1^Department of Prevention and Health Care, Wan Zhou District Zhong Gu Lou Agency Office Du Li Village Hospital, Chongqing 400010, China; ^2^Department of General Surgery, Wan Zhou District Shang Hai Hospital, Chongqing 400010, China

## Abstract

Gastric cancer (GC) is a common digestive tumor which ranks the fourth most common malignancy worldwide. Immunotherapy is a promising treatment for GC, especially for advanced gastric cancer (AGC). However, in clinical practice, not all patients are sensitive to immunotherapy. Recent studies showed that tumor mutation burden (TMB) is closely correlated with the response of immunotherapy. The current study identified a TMB-related genes' signature to predict the prognosis and immune feature of GC patients. Firstly, we acquired the TMB data and expression data from The Cancer Genome Atlas (TCGA) and the National Center for Biotechnology Information (NCBI) GEO databases. Then, we extracted TMB-related genes from the expression data of TCGA and two GEO cohorts. By using univariate Cox analysis, we identified that the 429 genes were correlated to GC patients' overall survival. Subsequently, an immune prognostic signature was constructed by using the least absolute shrinkage and selection operator analysis (LASSO) and multivariate Cox regression analysis. The signature could be utilized to predict the prognosis of GC patients. In addition, the signature showed a closed correlation with immune feature of GC patients. In conclusion, our risk signature could offer hints for the prognosis of GC patients and might provide insights to formulate new immunotherapy strategies for GC patients.

## 1. Introduction

Gastric cancer (GC) is one of the most common and aggressive tumors with over one million newly diagnosed cases and 768,000 deaths every year [1]. With the development of diagnosis and treatment of GC, the prognosis of patients has been improved. However, the overall survival of GC patients is still unsatisfactory. In particular, the 5-year overall survival rate of advanced gastric cancer is less than 5% [2]. The main reasons for poor survival of GC patients are tumor resistance to radiotherapy and chemotherapy and tumor immune escape. In recent years, immunotherapy has achieved the greatest improvement [3]. Immunotherapy such as immune checkpoint inhibitors (ICIs) could improve the overall survival of various cancers, including GC [4–7]. Clinical trials of Javelin Gastric 100, KEYNOTE-062, and Checkmate 649 demonstrated that immunotherapy has potential clinical application value [8–10]. However, in clinical application, the effective rate of the response to immunotherapy is only 20%. Therefore, this situation forced us to find effective biomarkers to predict the prognosis and immunotherapy response of GC.

Tumor mutation burden (TMB) was defined as the number of nonsynonymous somatic coding errors per megabase in cancer cells [11]. Mutations in driver genes might lead to the occurrence of tumors. However, mutation in tumor-specific neoantigens exerts a vital role in tumor-specific T cell-mediated antitumor immunity [12, 13]. Tumor cells will produce more new antigens with the increasing TMB level, and some of tumor-specific neoantigens could be recognized by immune cells, resulting in the death of tumor cells. Thus, TMB was utilized to predict the prognosis and immunotherapy response of tumors [14, 15]. TMB was proved to be effective in predicting the clinical benefit from immune checkpoint inhibitors in non-small-cell lung cancer and colorectal cancer [16, 17]. However, very little is known about the correlation between TMB-related genes and immune feature of GC.

In this study, we acquired TMB-related genes of GC from TCGA database. Three GC transcriptome cohorts were used to obtain and validate the prognostic function of the TMB-related genes' signature. The results demonstrated that the signature of TMB-related genes has predictive function in all three included GC cohorts. We also found that our signature is correlated with the immune characteristics of GC. In particular, the risk signature could be used to predict the potential immunotherapeutic benefit of GC patients.

## 2. Materials and Methods

### 2.1. Data Acquisition and Processing

The transcriptome sequence data of GC (FPKM) and corresponding clinical information were acquired from The Cancer Genome Atlas (TCGA) database (https://portal.gdc.cancer.gov/). The tumor mutation burden (TMB) data was downloaded from The Cancer Genome Atlas (TCGA) database (https://portal.gdc.cancer.gov/). Two GEO chips and their corresponding clinical information were obtained from the National Center for Biotechnology Information (NCBI) GEO database (https://www.ncbi.nlm.nih.gov/geo/). Two chips' accession numbers are GSE62254 and GSE84437, respectively. Patients with survival time more than 30 days were included. The genes with mutation over in 10 or more samples (3,569 genes) were selected for the construction of the signature.

### 2.2. Establishment of the Risk Signature

TCGA expression data was transformed into TPM. We intersected the TCGA cohort with GSE62254 and GSE84437 to obtain genes expressed in all three cohorts. Then, we extracted the genes with mutation over in 10 or more samples from three intersected expression matrix. A total of 2,891 TMB-related genes were screened for further analysis.

For the construction of the risk signature, we merged TCGA expression matrix and GSE62254 to acquire training set. Another GEO data GSE84437 was used as external validation set (testing set). Univariate Cox analysis was performed to identify genes with prognostic function in the training set. Then, LASSO regression analysis was conducted on 429 candidate prognostic TMB-related genes to obtain optimal candidates. A total of 29 genes were identified. Multivariate Cox analysis was utilized to construct the risk signature.

Risk score calculation formula is as follows:

(1) $$\begin{array}{c} \text{Risk score}\left(\text{patients}\right)=\overset{n}{\underset{k=1}{\sum }}\text{c}\text{oefficient }\left(\text{gene }k\right)*\text{expression}\left(\text{gene }k\right). \end{array}$$

In this formula, *n*, *k*, coefficient, and expression  represent for the number of selected gene, gene's number, coefficient value, and gene's expression value, respectively. Principal component analysis (PCA) and t-distributed stochastic neighbor embedding (t-SNE) were performed to visualize the efficiency of the signature in dimensionality reduction. R packages of “limma,” “sva,” “survival,” “survminer,” and “glmnet” were used in the above analyses.

### 2.3. Prognostic Function Exploration and External Validation of the Signature

All patients in training set and testing set were divided into two subgroups (high-risk group and low-risk group) according to the median risk score value. Kaplan-Meier analysis was performed on the training set and testing set to test the prognostic function of the risk signature. A time-dependent receiver operating characteristic curve was conducted to validate the accuracy of the risk signature in training set and testing set, respectively. The expression of genes in the signature was visualized using a heatmap.

Chi-square test was utilized to determine the correlation between the risk pattern and clinicopathological characteristics. Results were analyzed and visualized by using R packages of “limma,” “ggpur,” and “pheatmap.”

### 2.4. Independent Prognostic Function and Survival Prediction Function of the Signature

Univariate Cox analysis was utilized to determine the prognostic value of risk score and patients' clinicopathological characteristics. Multivariate Cox analysis was further performed to screen factors with independent prognostic function. A clinical related receiver operating characteristic curve was plotted to prove that risk score is the optimal factor to predict patients' survival.

A nomogram was constructed to predict patients' survival time by using factors with independent prognostic function. A calibration curve and a time-dependent receiver operating characteristic curve were used to assess the accuracy of the nomogram.

R packages of “survival,” “survivalROC,” “survminer,” “timeROC,” “rms,” and “regplot” were utilized in the above analyses.

Pathway enrichment and immune cell infiltration difference annotated by the signature.

Gene set enrichment analyses (GSEAs) were conducted to define KEGG enrichment differences between the high-risk group and the low-risk group. Results were visualized by using R package of “limma,” “http://org.hs.eg/.db,” “enrichplot,” and “clusterProfiler.” Immune cell infiltration statuses of GC patients were assessed by using R packages of “limma,” and “MCP counter.” Results were visualized by using R packages of “limma” and “ggpubr.”

### 2.5. Immune Pathway Difference and Immunotherapy Response Prediction

The single-sample gene set enrichment analysis (ssGSEA) method was utilized to evaluate the difference of immune-related pathways between the high-risk group and the low-risk group. R packages of “limma,” “GSVA,” “GSEABase,” “ggpubr,” and “reshape2” were used in above analysis.

The expression difference of HLA-related genes and immune checkpoint genes between two groups of patients was determined by using “limma,” “ggplot2,” “reshape2,” and “ggpubr” packages of R. Tumor Immune Dysfunction and Exclusion (TIDE) score was acquired from http://tide.dfci.harvard.edu. Pearson correlation test was used to evaluate the correlation relation between risk score and TIDE prediction score. The immunotherapy response difference between high-risk and low-risk groups of patients was evaluated by using “limma” and “ggpubr” package of R.

### 2.6. Statistical Analysis

All data were analyzed by using the R (version 4.1.0) software. Survival differences were determined by using the Kaplan-Meier analysis. Correlation between risk pattern and clinical characteristics was determined by using chi-square test. Pearson correlation test was used to evaluate the correlation coefficient.

## 3. Results

### 3.1. Establishment of the TMB Gene-Related Signature in GC

Tumor mutation burden (TMB) is strongly associated with gastric cancer (GC). To identify TMB-related genes in GC, we obtained the TMB data of GC and acquired genes with mutation more than in 10 samples. A total of 3,569 genes were identified. To further acquire the expression data of these 3,569 genes, we obtained the expression data from The Cancer Genome Atlas (TCGA) and the National Center for Biotechnology Information (NCBI) GEO databases. Patients with survival time less than 30 days were excluded (including 305 samples from TCGA, 300 samples from GSE62254, and 431 samples from GSE84437). After normalizing and intersecting the expression data from two databases, 2,891 out of 3,569 TMB-related genes were screened for further studies.

**Figure 1:**
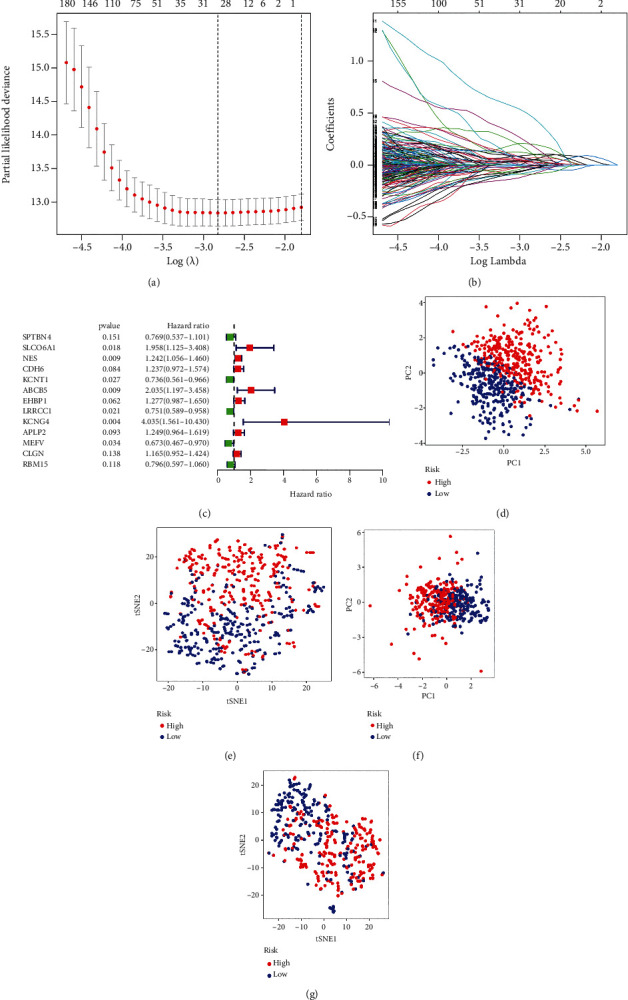
Establishment of the risk signature. (a, b) LASSO regression analysis was used to screen the prognostic optimal candidate genes. (c) Multi-Cox analysis was conducted for the construction of the risk signature. (c–g) PCA and t-SNE analyses were used to visualize the dimensionality reduction annotated by the risk signature.

To obtain the TMB-related genes' risk signature associated with the prognosis of all GC patients, we constructed the risk signature by applying TCGA cohort and GSE62254 cohort (training set) and validated the performance of the risk signature in GSE84437 cohort (testing set). Univariate Cox analysis was performed to screen prognostic TMB genes. 429 out of 2,891 genes were acquired (Supplementary table 1). 429 candidate prognostic TMB genes were subjected to 1,000 times LASSO regression analysis, and 29 best candidates were obtained (Figures 1(a) and 1(b) and Supplementary table 2). Then, we conducted multivariate Cox analysis on these 29 genes to construct the risk signature. A total of 13 genes (*SPTBN4*, *SLCO6A1*, *NES*, *CDH6*, *KCNT1*, *ABCB5*, *EHBP1*, *LRRCC1*, *KCNG4*, *APLP2*, *MEFV*, *CLGN*, and *RMB15*) were identified in the risk signature (Figure 1(c) and Supplementary table 3). According to the median value of risk score, all patients were divided into the high-risk group and the low-risk group. Based on the risk pattern of GC patients, principal component analysis (PCA) and t-distributed stochastic neighbor embedding (t-SNE) were used for dimensionality reduction of the genes. We observed that our risk signature has an elevated efficiency in distinguishing the high-risk and low-risk patients, both in training set and testing set (Figures 1(d)–1(g)).

### 3.2. Predictive Performance of the Signature

**Figure 2:**
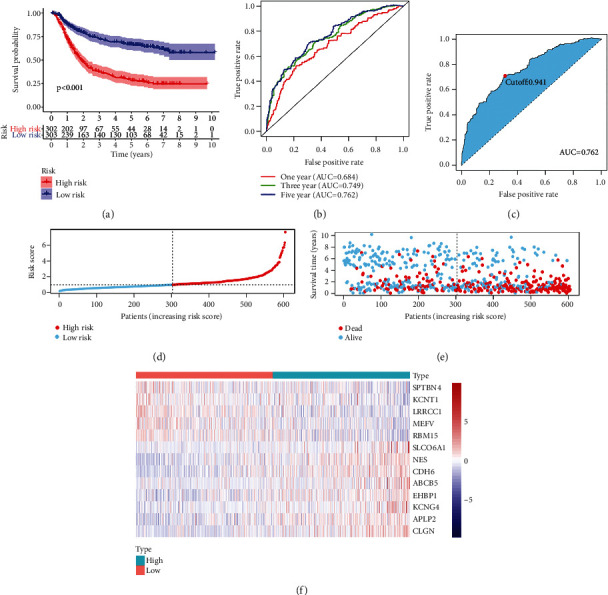
(a) Predictive function of the risk signature in training set. Kaplan-Meier analysis was conducted to determine survival difference between the high-risk group and the low-risk group. (b, c) Receiver operating characteristic (ROC) curve was plotted to assess the accuracy of the signature in predicting patients' survival outcomes. (d, e) Patients were ranked according to risk score value, and the survival status of the patients was visualized. (f) A heatmap was plotted to visualize the expression pattern difference of the genes in the signature between the high-risk group and the low-risk group.

In order to test the prognostic function of the signature in training set, we first constructed a survival curve and observed that the survival probability of high-risk patients was lower than that in low-risk patients. (Figure 2(a)). The predictive accuracy of the signature was tested using a time-dependent curve of the ROC. The area under the curve (AUC) values for each ROC curve were 0.684, 0.749, and 0.762, at one year, three years, and five years, respectively (Figure 2(b)). The median cut-off value of the five years' ROC curve is 0.941 (Figure 2(c)). According to the median risk score of GC patients, we ranked all patients and analyzed their distribution (Figure 2(d)). We found that mortality was higher in the high-risk group than in the low-risk group. With the increasing of risk score, the number of death patients also increased (Figure 2(e)). Among 13 genes in the signature, five genes (*SPTNN4*, *KCNT1*, *LRRCC1*, *MEFV*, and *RMB15*) were downregulated in the high-risk group. Another seven genes (*SLCO6A1*, *NES*, *CDH6*, *ABCB5*, *EHBP1*, *KCNG4*, *APLP2*, and *CLGN*) were upregulated in the high-risk group (Figure 2(f)).

**Figure 3:**
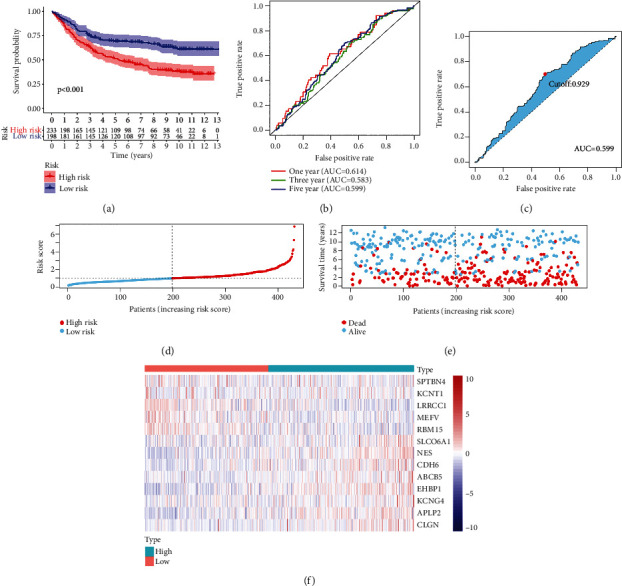
(a) Predictive function of the risk signature in testing set. Kaplan-Meier analysis was conducted to determine survival difference between the high-risk group and the low-risk group. (b, c) Receiver operating characteristic (ROC) curve was plotted to assess the accuracy of the signature in predicting patients' survival outcomes. (d, e) Patients were ranked according to risk score value, and the survival status of the patients was visualized. (f) A heatmap was plotted to visualize the expression pattern difference of the genes in the signature between the high-risk group and the low-risk group.

To further validate the function of the signature, we also test the predictive performance of the signature in the testing group. Kaplan-Meier analysis indicated that patients with higher risk score have worse survival outcomes than patients in the low-risk group (Figure 3(a)). AUC values of the time-dependent ROC were 0.614, 0.583, and 0.599, at one year, three years, and five years, respectively (Figure 3(b)). The median cut-off value of the five years' ROC curve is 0.929 (Figure 3(c)). After ranking all patients according to the median value of risk score (Figure 3(d)), we also found that the mortality in the high-risk score group is higher than the low-risk group (Figure 3(e)). Thirteen genes between the high-risk group and the low-risk group exhibited a similar expression pattern in the training set (Figure 3(f)). These results suggested that our signature could be applied to predict the prognosis of GC patients.

### 3.3. Clinical Evaluation by Using the Risk Signature

**Figure 4:**
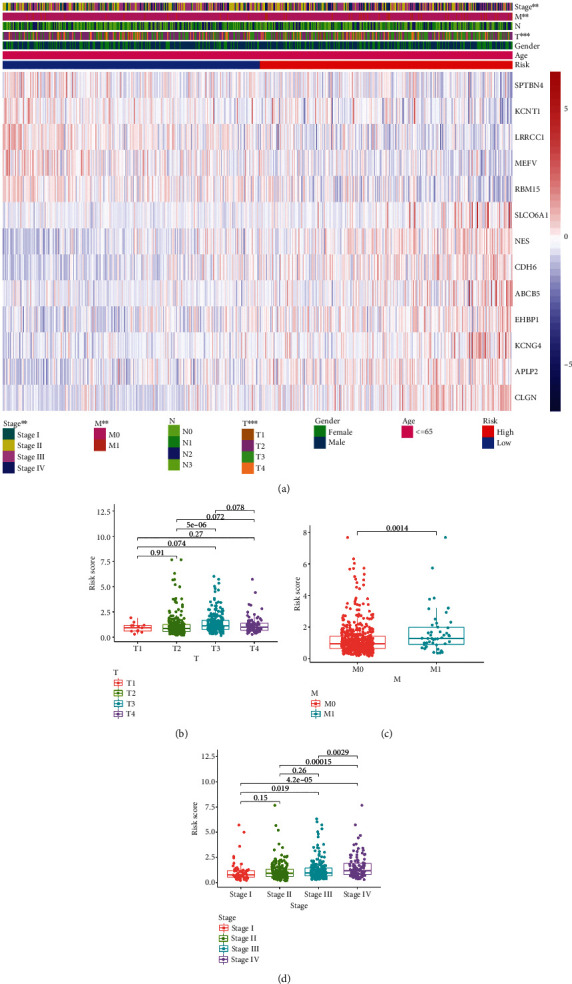
Relationship between risk pattern and clinicopathological characteristics of GC patients. (a) A heatmap was used to show the correlation between risk pattern and all clinicopathological characteristics of GC patients. (b) Correlation between risk score and tumor stage of GC patients. (c) Correlation between risk score and metastasis stage of GC patients. (d) Correlation between risk score and clinic stage of GC patients.

To detect the relationship between the risk pattern and clinicopathological characteristics of GC patients, we conducted chi-square test and found that risk score was significantly correlated with tumor stage, metastasis stage, and clinical stage (Figure 4(a)). Patients with tumor stage III have a higher risk score (Figure 4(b)). Patients with metastasis have a higher risk score than that of patients without metastasis (Figure 4(c)). In addition, we also observed that with an increasing of clinical stage, patients with higher clinical stage have higher risk score (Figure 4(d)).

In clinical application of the prognostic model, we often encountered patients with various clinical characteristics. To further explore the clinical application value of the signature, we divided the patients into two groups according to different clinical characteristics and analyzed the difference of survival outcomes between low-risk patients and high-risk patients in all subgroups. Interestingly, we observed that in all subgroups, the survival prognosis of high-risk patients was worse than that of low-risk patients (Supplementary Figure 1), indicating that our risk signature is effective in predicting the prognosis of patients with various clinical characteristics.

### 3.4. Independent Prognostic and Survival Time Predictive Value of the Risk Signature

**Figure 5:**
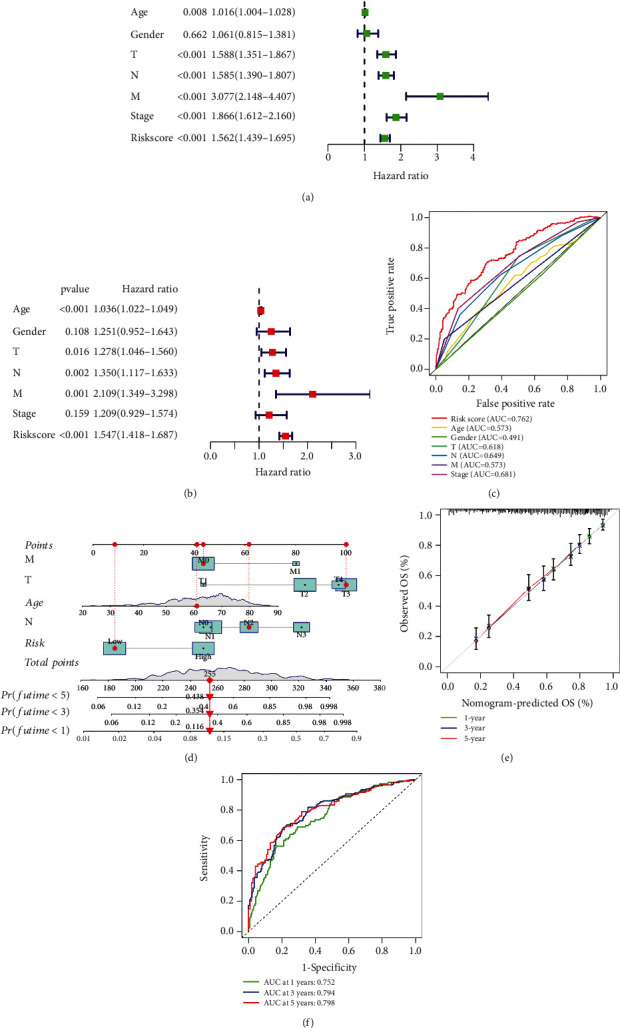
Independent prognostic and survival time predictive value of the risk signature. (a, b) Univariate Cox analysis and multivariate Cox analysis were utilized to evaluate the independent prognostic value of the signature. (c) Receiver operating characteristic curve was plotted to prove the superiority of the risk score in predicting patient's survival than clinical characteristics. (d) A nomogram was constructed to predict patients' survival time by using indicators with independent prognostic function. (e, f) A calibration curve and a ROC curve were plotted to test the accuracy of the nomogram at 1, 3, and 5 years, respectively.

Kaplan-Meier analysis demonstrated that our risk signature has undeniable value in predicting the prognosis of GC patients. In order to further prove that the prognostic function of the risk signature is not related to the clinical characteristics of the patients, we performed univariate Cox analysis and multivariate Cox analysis and found that risk score could be used as an independent prognostic indicator (Figures 5(a) and 5(b)), which further proved the prognostic value of our signature. In addition, AUC value of ROC curve indicated that risk score is superior to other clinical characteristics in predicting the prognosis and survival of patient (Figure 5(c)). To better explore the prognostic value of the risk signature, we combined risk score and significant clinical characteristics in multivariate Cox analysis (including M stage, T stage, age, and N stage) and constructed a nomogram to predict GC patients' survival time (Figure 5(d)). The accuracy of the nomogram was determined by calibration curves and ROC curves. The calibration curves showed that the overall survival (OS) time predicted by the nomogram is almost the same as the observed OS time (Figure 5(e)). Furthermore, AUC value of ROC curves was 0.752, 0.794, and 0.798, at one year, three years, and five years, respectively (Figure 5(f)).

### 3.5. Association between the Risk Signature and Immune Feature of GC Patients

**Figure 6:**
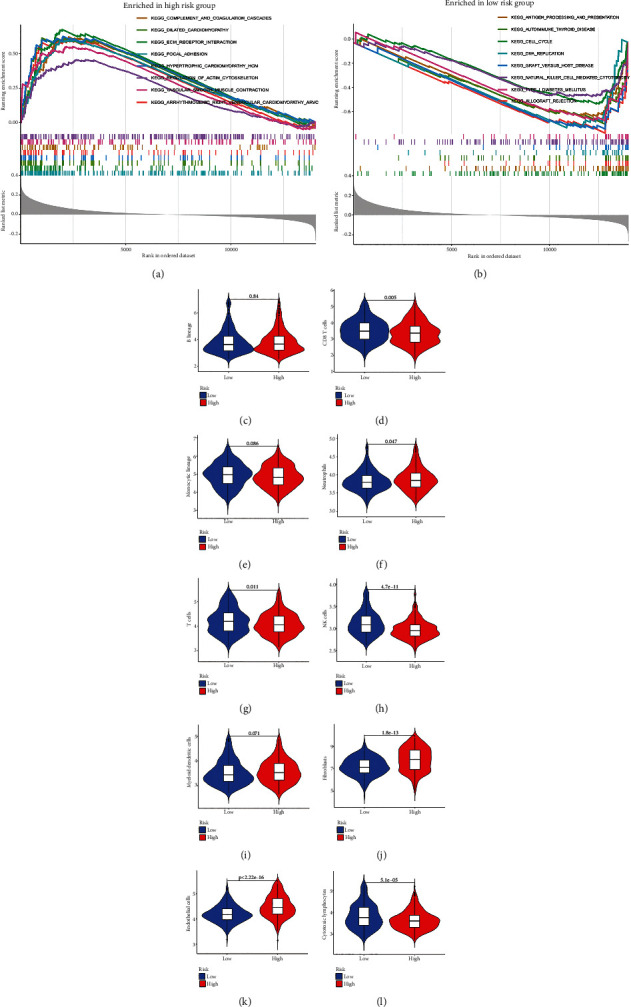
Immune feature differences between the high-risk group and the low-risk group. (a, b) GSEA was used to evaluate the difference in the enrichment of KEGG between the high-risk group and the low-risk group. (c–l) MCP counter was utilized to evaluate immune cell infiltration difference between the high-risk group and the low-risk group.

Consider that TMB is closely correlated with immune feature especially in the response to immunotherapy. To determine whether TMB-related genes' signature could separate patients into two subgroups with different features, we compared the differences in the enrichment of the pathways between the high-risk group and the low-risk group by using GSEA. Interestingly, we observed that immune-related pathways such as antigen processing and presentation, autoimmune thyroid disease, graft versus host disease, natural killer cell-dediated cytotoxicity, and allograft rejection were enriched in the low-risk group (Figures 6(a) and 6(b)), which suggested that the low-risk group might have better immune function in response to tumor. Apart from this, we also detected the immune infiltration differences between low-risk and high-risk patients by using R package of “MCP counter.” We found that low-risk group patients have a more infiltration of CD8+ T cells, NK cells, T cells, and cytotoxic lymphocytes. However, low-risk group patients have less infiltration of neutrophils, fibroblasts, and endothelial cells (Figures 6(c)–6(l)). These results suggested our risk signature is closed correlated with the immune feature of GC patients.

### 3.6. Correlation between the Risk Signature and Potential Benefit of Immunotherapy

**Figure 7:**
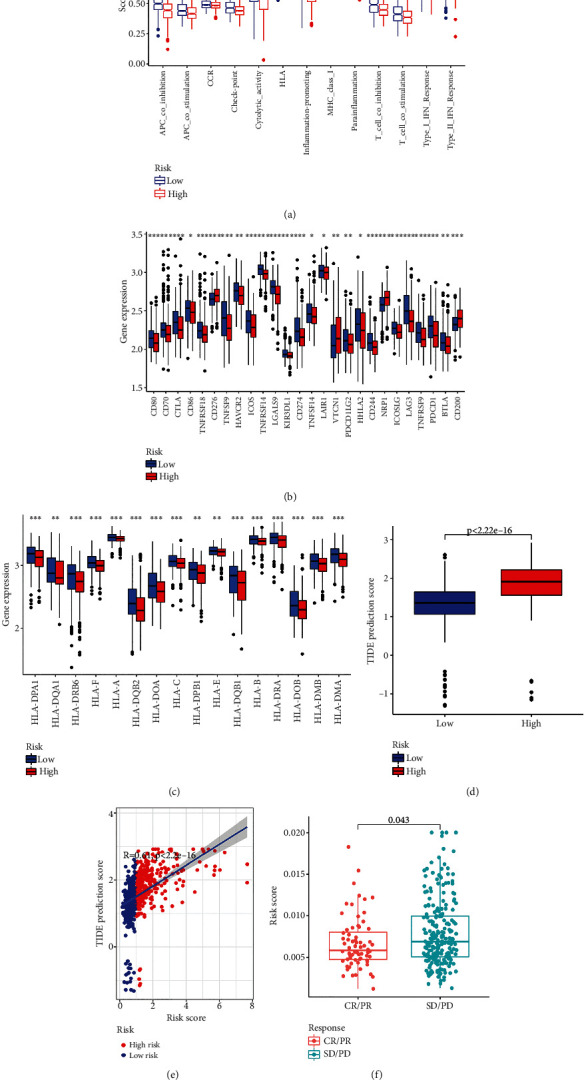
Predicting immunotherapy response by using the risk signature. (a) Differences in the enrichment of immune-related pathways were determined by using ssGSEA (^∗∗^*P* < 0.01 and ^∗∗∗^*P* < 0.001). (b) Expression level difference of immune checkpoint genes between the low-risk group and the high-risk group (^∗^*P* < 0.05, ^∗∗^*P* < 0.01, and ^∗∗∗^*P* < 0.001). (c) Expression difference of HLA-related genes between the low-risk group and the high-risk group (^∗^*P* < 0.05, ^∗∗^*P* < 0.01, and ^∗∗∗^*P* < 0.001). (d) TIDE predicting score was acquired to predict immunotherapy response of GC patients. (e) Correlation between TIDE predicting score and risk score. (f) External validation set (IMvigor210) was used to determine the function of the signature in predicting patients' immunotherapy response.

To better understand the immune feature differences between low-risk patients and high-risk patients, we further compared the difference of 13 immune-related pathways between the high-risk group and the low-risk group by using the ssGSEA method. The result demonstrated that 11 of the 13 pathways have higher activities in the low-risk group, whereas other two pathways exerted no difference between the two groups (Figure 7(a)). In addition, it is reported that patients with higher levels of TMB could produce more proteins that could be recognized by the immune system. Immune cells are more likely to recognize and eliminate those tumor cells with high TMB. Based on this, we speculated that the low-risk group might have a better immunotherapy response than the high-risk group. The expression of immune checkpoint genes and HLA-related genes was closely associated with immunotherapy response [18, 19]. Thus, we evaluated the expression of immune checkpoint genes and HLA-related genes between two groups. As expected, we observed that most of the immune checkpoint genes and all 16 HLA-related genes have a higher expression level in the low-risk group (Figures 7(b) and 7(c)). In addition, we also acquired the Tumor Immune Dysfunction and Exclusion (TIDE) score and compared the difference in TIDE score between two groups. Results indicated that the TIDE score of patients in the high-risk group was relatively higher, indicating that the immunotherapy response of patients in the high-risk group was poor (Figure 7(d)). There was a significant positive correlation between risk score and TIED prediction score (Figure 7(e)). To extend our findings, we acquired a cohort treated with ICI (IMvigor210 cohort) and validated the function of our signature in predicting immunotherapy response. Results also indicated that patients with progression disease (PD) and stable disease (SD) had higher risk scores (Figure 7(f)). All these evidences suggested that our risk signature could be used to predict the potential benefits of immunotherapy.

## 4. Discussion

Gastric cancer (GC) is one of the most common malignancies, which is characterized by poor prognosis. According to the Global Cancer Statistics 2020, GC is the fourth most common cause of cancer mortality [1]. The main reasons for poor survival of GC patients are the resistance of tumor cells to radiotherapy and chemotherapy and tumor cell immune escape. With the development of the treatment in GC, emerging treatment strategies, especially immunotherapy, have been widely used in GC patients. The clinical trials of Javelin Gastric 100, KEYNOTE-062, and Checkmate 649 have proved that first-line treatment of immunotherapy could improve the prognosis of some GC patients [8–10]. However, the effective rate of the response to immunotherapy is only 20%. Consider the poor prognosis and the potential benefit of immunotherapy. It is urgent to find effective biomarkers to predict the prognosis and immunotherapy response of GC patients.

Tumor mutation burden (TMB) is defined as the number of nonsynonymous somatic coding errors per megabase in cancer cells [11]. Increasing evidences indicated TMB could be used to predict patients' prognosis. It is reported that higher levels of TMB are associated with better survival outcomes in numerous tumors but worse prognosis in a few tumors [20, 21]. The levels of TMB are also correlated with the prognosis of GC patients. The increasing TMB level indicates a good prognosis of GC patients [22–24]. In addition, TMB could be used to predict the therapy response of patients to immunotherapy in multiple cancer types [25–27]. A recent study revealed that TMB might promote infiltration of immune cells [28], which indicated that TMB is associated with immune feature of GC. However, there are few studies focus on the function of TMB-related genes in GC. Further researches on the correlation between TMB-related genes and immune feature of GC will be helpful to identify the critical predictive biomarkers.

To explore the correlation between TMB-related genes and GC, we first acquired TMB-related genes from TMB data of The Cancer Genome Atlas (TCGA) database. Then, the expression matrix of TCGA, GSE62254, and GSE84437 was intersected. A total of 2,891 TMB-related genes were identified in these three cohorts. We constructed a predictive signature using TCGA cohort and GSE62254 cohort (training set). Another cohort GSE84437 was used as an external validation set to prove the prediction function of the risk signature. Through various methods, we constructed a risk signature using 13 TMB-related genes (*SPTBN4*, *SLCO6A1*, *NES*, *CDH6*, *KCNT1*, *ABCB5*, *EHBP1*, *LRRCC1*, *KCNG4*, *APLP2*, *MEFV*, *CLGN*, and *RMB15*). Interestingly, we only found the expression of two genes was reported to be correlated with GC. *CDH6* was reported to be associated with tumor progression and poor prognosis of GC [29]. *ABCB5* was identified as hub gene which correlated with the pathogenesis and prognosis of GC [30]. However, the expression of other 11 genes has not been reported to be related with GC. We speculated the main reason is that the genes in the signature were screened from TMB-related genes, not from the differentially expressed genes between normal tissues and tumor tissues of GC patients. After acquiring the risk signature, we tested the predictive function of the risk signature. Results demonstrated that our signature could be used to predict the prognosis of GC patients in both the training set and the testing set.

TMB can affect the degree of immune infiltration and response of immunotherapy in a variety of cancers [31–33]. In order to further explore the deeply mechanism mediated by our risk signature, we conducted the GSEA to determine the differences in the enrichment of the pathways between the high-risk group and the low-risk group. We found that immune-related pathways such as antigen processing and presentation, autoimmune thyroid disease, graft versus host disease, natural killer cell-dediated cytotoxicity, and allograft rejection were enriched in the low-risk group, which indicated that these two groups have different immune feature. Thus, we further determined the difference of immune cell infiltration between two groups. We observed that low-risk group patients have a more infiltration of CD8+ T cells, NK cells, T cells, and cytotoxic lymphocytes. However, low-risk group patients have a less infiltration of neutrophils, fibroblasts, and endothelial cells. High infiltration of CD8+ T cells, NK cells, and cytotoxic lymphocytes was reported to be associated with better survival outcomes in patients with cancers [34–37]. High neutrophil infiltration of immune cells indicated a higher malignancy and a worse prognosis of pancreatic ductal adenocarcinoma and hepatocellular carcinoma [38, 39]. Infiltration of neutrophils could also promote migration and invasion of gastric cancer cells via EMT pathway [40]. Fibroblasts are associated with poor prognosis and could enhance tumor progression in various tumors including GC [41–43]. Endothelial cells were also reported to be associated with poor prognosis of GC and could promote glioma cell migration [43, 44]. These results further supported the application of our risk model as a biomarker in predicting the prognosis of GC and suggested that our risk signature is closely correlated with the immune feature of GC patients.

Our above results indicated the risk signature could divide patients into two subgroups with different immune feature. To explore whether our signature could also exert function in predicting immunotherapy response, we compared the expression of HLA-related genes and immune checkpoint genes between the low-risk patients and the high-risk patients. Patients with higher expression of HLA-related genes and immune checkpoints might have a better response to immune checkpoint blockade [19, 45]. We found that expression of most HLA-related genes and immune checkpoints genes was higher in the low-risk group, indicating a better immunotherapy response. We also obtained the immunotherapy data from TICA to validate our results. Results demonstrated that patients in the low-risk group might have a better immunotherapy response, which is consistent with our findings. In addition, we acquired the immunotherapy data of an external cohort treated with ICI (IMvigor210 cohort) and validated that our signature could be used to predict immunotherapy response of tumor patients.

In conclusion, our findings indicated that the TMB-related genes' signature has a predictive function in GC patients. The risk signature could also be used to predict the immune feature especially for the immunotherapy of GC patients, which might provide valuable clues for the development of immunotherapy in GC.

## Data Availability

TCGA cohort data and TMB data generated for this study can be found in the TCGA database (https://cancergenome.nih.gov/). The GEO datasets generated for this study can be found in the GEO-GSE62254 and GEO-GSE84437 (https://www.ncbi.nlm.nih.gov/geo/).

## Supplementary Materials

Supplementary 1 Figure 1: survival analyses were conducted in patients with different ages (A and B), genders (C and D), T stages (E and F), N stages (G and H), M stage (I and J), and clinical stage (K and L).

Supplementary 2 Table 1: result of univariate Cox analysis. Supplementary Table 2: 29 optimal candidates acquired from LASSO regression analysis. Supplementary Table 3: result of multivariate Cox analysis.
