# Supplementary material for: Significance of a Tumor Mutation Burden Gene Signature with Prognosis and Immune Feature of Gastric Cancer Patients
Source: Int J Genomics. 2022 Jun 8;2022:7684606. doi: 10.1155/2022/7684606 (PMC9201710; doi:10.1155/2022/7684606)
Supplement: Supplementary 1 — Figure 1: survival analyses were conducted in patients with different ages (A and B), genders (C and D), T stages (E and F), N stages (G and H), M stage (I and J), and clinical stage (K and L). [file 7684606.f1.pdf]

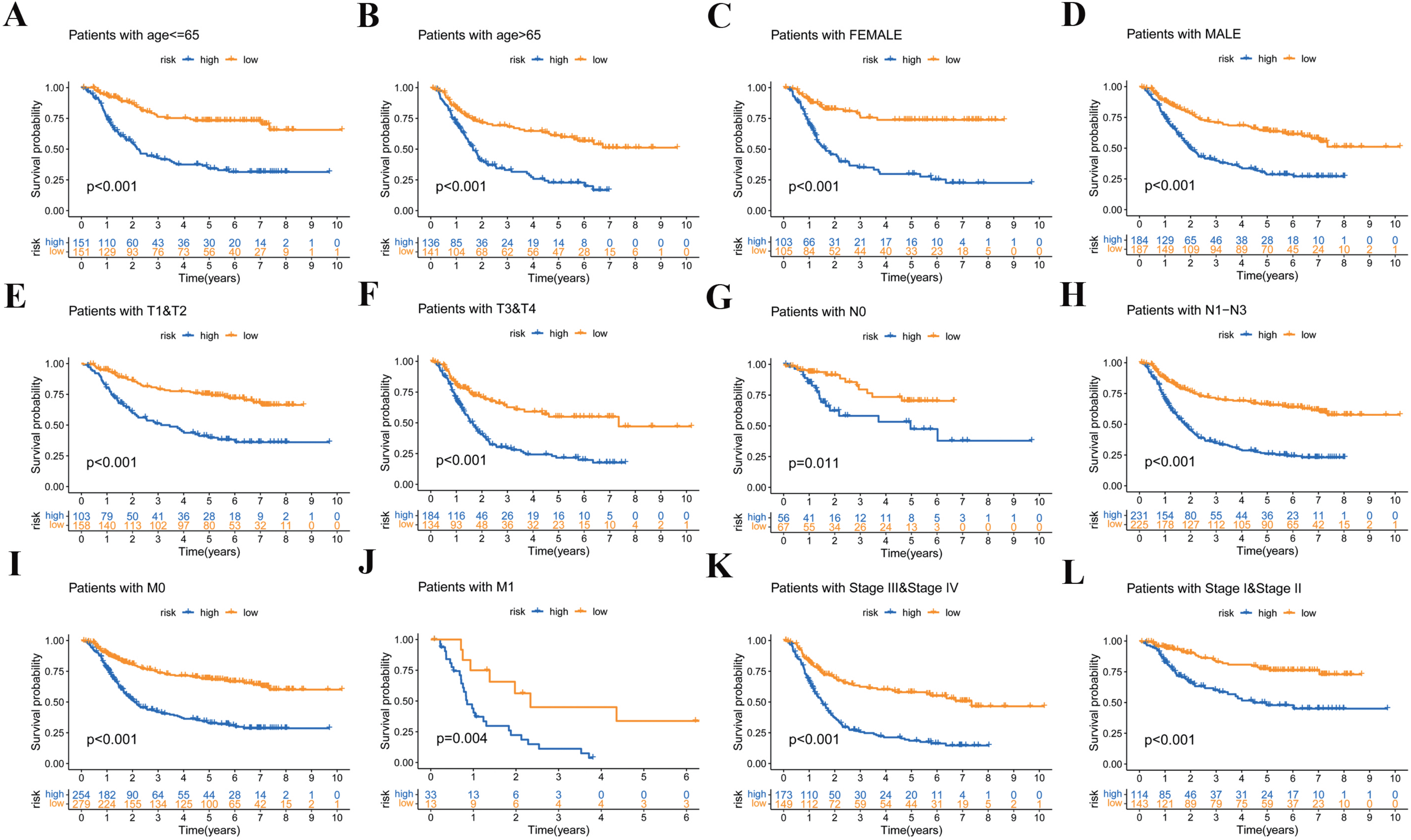
A
Patients with age<=65
risk high low
Survival probability
p<0.001
risk high 151 110 60 43 36 30 20 14 2 1 0
low 151 129 93 76 73 56 40 27 9 1 1
Time(years)
B
Patients with age>65
risk high low
Survival probability
p<0.001
risk high 136 85 36 24 19 14 8 0 0 0 0
low 141 104 68 62 56 47 28 15 6 1 0
Time(years)
C
Patients with FEMALE
risk high low
Survival probability
p<0.001
risk high 103 66 31 21 17 16 10 4 1 1 0
low 105 84 52 44 40 33 23 18 5 0 0
Time(years)
D
Patients with MALE
risk high low
Survival probability
p<0.001
risk high 184 129 65 46 38 28 18 10 1 0 0
low 187 149 109 94 89 70 45 24 10 2 1
Time(years)
E
Patients with T1&T2
risk high low
Survival probability
p<0.001
risk high 103 79 50 41 36 28 18 9 2 1 0
low 158 140 113 102 97 80 53 32 11 0 0
Time(years)
F
Patients with T3&T4
risk high low
Survival probability
p<0.001
risk high 184 116 46 26 19 16 10 5 0 0 0
low 134 93 48 36 32 23 15 10 4 2 1
Time(years)
G
Patients with N0
risk high low
Survival probability
p=0.011
risk high 56 41 16 12 11 8 5 3 1 1 0
low 67 55 34 26 24 13 3 0 0 0 0
Time(years)
H
Patients with N1−N3
risk high low
Survival probability
p<0.001
risk high 231 154 80 55 44 36 23 11 1 0 0
low 225 178 127 112 105 90 65 42 15 2 1
Time(years)
I
Patients with M0
risk high low
Survival probability
p<0.001
risk high 254 182 90 64 55 44 28 14 2 1 0
low 279 224 155 134 125 100 65 42 15 2 1
Time(years)
J
Patients with M1
risk high low
Survival probability
p=0.004
risk high 33 13 6 3 0 0 0
low 13 9 6 4 4 3 3
Time(years)
K
Patients with Stage III&Stage IV
risk high low
Survival probability
p<0.001
risk high 173 110 50 30 24 20 11 4 1 0 0
low 149 112 72 59 54 44 31 19 5 2 1
Time(years)
L
Patients with Stage I&Stage II
risk high low
Survival probability
p<0.001
risk high 114 85 46 37 31 24 17 10 1 1 0
low 143 121 89 79 75 59 37 23 10 0 0
Time(years)
